# Benchmarking Machine Learning Algorithms for Microbial Electromethanogenesis: A Comprehensive Assessment with SHapley Additive exPlanation-Based Insights

**DOI:** 10.1021/acssuschemeng.5c09770

**Published:** 2025-12-16

**Authors:** Siddharth Gadkari, Raphael Souza de Oliveira, Silvia Bolognesi, Sebastià Puig, Erick Giovani Sperandio Nascimento

**Affiliations:** a School of Chemistry and Chemical Engineering, 3660 University of Surrey, Guildford GU2 7XH, United Kingdom; b Institute for Sustainability, 3660 University of Surrey, Guildford GU2 7XH, United Kingdom; c Stricto Sensu Department, 306313 SENAI CIMATEC University, Salvador, Bahia 41650-010, Brazil; d LEQUiA. Institute of the Environment, 16738 University of Girona, Campus Montilivi. C/Maria Aurelià Capmany, 69, Girona E-17003, Spain; e Surrey Institute for People-Centred Artificial Intelligence, Faculty of Engineering and Physical Sciences, 3660 University of Surrey, Guildford GU2 7XH, United Kingdom; f Global Centre for Clean Research (GCARE), School of Sustainability, Civil and Environmental Engineering and Physical Sciences, 3660 University of Surrey, Guildford GU2 7XH, United Kingdom

**Keywords:** microbial electromethanogenesis, biogas upgrading, machine learning, convolutional neural network, SHAP analysis

## Abstract

Microbial electromethanogenesis (EM) presents a promising pathway for sustainable biogas upgrading, but accurately predicting its performance is challenging due to complex, nonlinear process dynamics. Here, we systematically compared seven supervised machine learning (ML) algorithms, including one-dimensional convolutional neural network (1D-CNN), multilayer perceptron (MLP), gradient boosting regressor (GBR), adaptive boosting regressor (AdaBoost), stacking regressors, and K-nearest neighbors (kNN), for their predictive biomethane production capabilities using experimental data from EM bioelectrochemical systems (EM-BESs). The data set encompassed operational parameters such as optical density (OD_600_), pH, electrical conductivity (EC, mS/cm), average applied current (A m^–2^), and CO_2_ availability (mol). After hyperparameter optimization, the 1D-CNN model exhibited superior predictive performance (*R* ^2^ = 0.934), significantly outperforming traditional ML methods. To move beyond prediction and uncover mechanistic insights, a feature importance analysis was conducted on the CNN model using SHapley Additive exPlanations (SHAP). The analysis revealed that average current, OD_600_, and pH were the most influential features in biomethane production, confirming that the model learned relationships grounded in fundamental bioelectrochemical principles. The SHAP analysis also identified complex, nonmonotonic effects of other variables, providing deeper process understanding. This study not only demonstrates the promising ability of ML, especially deep learning architectures, to advance EM optimization but also provides mechanistic insights into the factors governing bioelectrochemical methanogenesis. These findings are broadly applicable to analogous BESs, particularly microbial electrosynthesis (i.e., commodity chemical) and microbial electrolysis cells (i.e., biohydrogen), offering potential for enhancing system performance through data-driven operational control across sustainable biotechnology applications.

## Graphical abstract

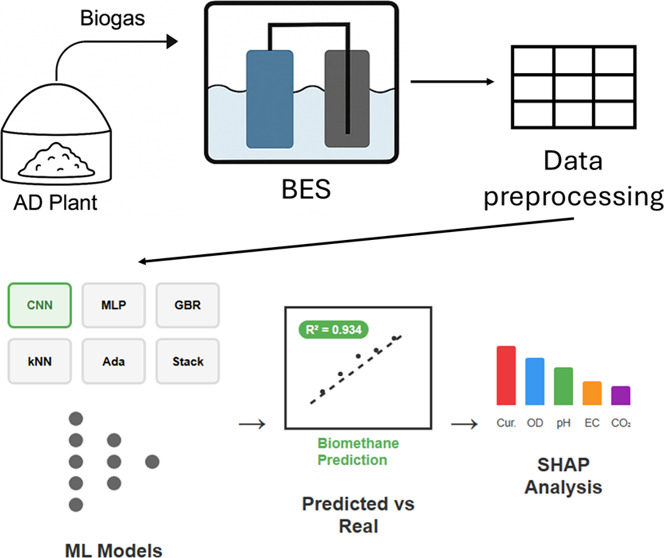

## 1 Introduction

Biomethane production via anaerobic digestion (AD) represents a versatile and effective approach to both CO_2_ reduction and renewable energy generation.^1^ Methane is a versatile fuel, compatible with the existing natural gas infrastructure, it is easy to store, and transport compared to other fuels (among all, green hydrogen), and efficient to distribute.^2^ However, a significant limitation of conventional AD lies in the composition of the produced biogas, where residual CO_2_ content accounts for approximately 40% of its composition, substantially reducing its energy density and limiting its direct utilization as a natural gas substitute.^3^ This necessitates biogas upgrading processes to increase methane content and enhance energy value. While various upgrading technologies exist, microbial electrochemical technologies (METs) have emerged as particularly promising approaches, offering a sustainable pathway to not only remove CO_2_ from biogas but simultaneously convert it into additional methane, thereby maximizing energy recovery.^4^ Within METs, microbial electromethanogenesis (EM) is a novel power-to-gas technology in which methanogens at the biocathode convert CO_2_ to CH_4_ using electrons supplied from an external power source.^5^ This approach offers a dual advantage: CO_2_ removal from biogas streams coupled with additional methane generation, effectively addressing both the purification and energy enhancement needs of biogas upgrading.^6^ Because the process operates at mesophilic temperature, near-ambient pressure, and uses microbial catalysts instead of expensive chemicals or noble metals, EM offers an attractive, low-cost alternative to abiotic electrocatalytic methanation and other upgrading technologies.^6,7^

Successful EM operation requires precise control of multiple interacting operational parameters. Applied voltage/current and pH (ideally 6.5–8) are critical factors, with sufficient buffer capacity needed to support methanogenic activity.^8^ Additional important parameters include biofilm maturity, conductivity, CO_2_ availability, electrode architecture, membrane properties, temperature, and reactor configuration. While EM has demonstrated promising performance at laboratory-to-pilot scale, achieving CO_2_ conversion rates exceeding 90% and production rates up to 212.5 L CH_4_ m^–2^ d^–1^, scaling and sustaining such performance in complex, real-world environments remain challenging.^9^ Recently, a galvanostatically controlled 17 L EM-BES achieved the highest reported methane production rate to date (280 L CH_4_ m^–3^ d^–1^) at an energy efficiency of 40%.^10^ Nevertheless, sustaining such performance under complex operational conditions is hindered by the intricate interdependencies between operational variables can result in suboptimal or unstable system behavior if not precisely controlled. Optimizing this multidimensional parameter space through trial-and-error experimentation alone is time-consuming and costly, necessitating more systematic, computational approaches for process optimization and scale-up.^11,12^

To address these optimization challenges, researchers have traditionally employed first-principles-based mathematical models to investigate key variables and their interactions in greater detail. Over the past decade, several mathematical models have been developed for different bioelectrochemical systems (BESs), including EM, contributing to system optimization efforts.^11,13^ These deterministic models describe BES phenomena using differential and algebraic equations, requiring comprehensive process understanding for accurate implementation. However, the complexity of BES stems from numerous unknown interdependencies between parameters, which fundamentally limits the accuracy of conventional deterministic models in predicting performance metrics. While certain phenomena such as material balance and current distribution can be described using established governing laws, others, particularly microbial kinetics and their dependence on charge balance and potential, remain poorly understood and difficult to model accurately. Biofilm kinetics further depend on extracellular electron transfer (EET) mechanisms from the cathode to microorganisms, processes that are not fully elucidated. These knowledge gaps prevent rigorous model formalization through established mathematical relationships, rendering even the most sophisticated deterministic models as approximations with inherent limitations.^14^

To overcome these limitations, machine learning (ML) techniques present a powerful data-driven approach that has gained increasing adoption in bioprocess applications. Unlike their deterministic counterparts, ML algorithms excel by learning complex relationships directly from experimental data, without necessitating explicit, predefined knowledge of all governing laws and equations. This capability makes ML particularly well-suited for BES applications where mechanistic understanding remains incomplete.

The utility of ML in BES has been demonstrated across various applications.^13,15^ In microbial fuel cells (MFCs), for instance, models such as Artificial Neural Networks (ANNs), Support Vector Regression (SVR), and ensemble methods like XGBoost have been successfully used to predict power density and output voltage with high accuracy (e.g., 99% in some studies, or error percentages as low as 0.5% for power generation predictions).^15,16^ Time-series forecasting using LSTMs has also proven effective for predicting MFC energy generation.^17^ For microbial electrolysis cells (MECs) and broader microbial electrosynthesis (MES) applications, Random Forest (RF) models have predicted hydrogen production with high R^2^ values (>0.92), and XGBoost has accurately forecasted acetate and ethanol yields (R^2^ of 0.877 for acetate, 0.727 for ethanol.^18,19^ This consistent success across analogous technologies strongly suggests the untapped potential of ML for advancing EM processes. However, while these studies confirm the utility of established ensemble methods for various BES applications, the optimal ML architecture for the unique complexities of EM remains an open question. Specifically, it is unclear whether advanced deep learning models, such as Convolutional Neural Networks (CNNs), could offer superior predictive power. CNNs, with their inherent ability to learn hierarchical patterns and local feature interactions, may be uniquely suited to deciphering the complex, synergistic effects of electrochemical, biological, and chemical parameters that govern EM performance, a hypothesis that has not been rigorously tested in this domain.

Furthermore, data sets from BESs, EM included, are often characterized by limited sample sizes, inherent variability due to the complex interplay of biological and electrochemical factors, and potential inconsistencies arising from diverse experimental setups and measurement protocols.^20^ Developing robust and generalizable predictive models from such inherently ‘messy’ data remains a significant challenge. Addressing this requires a methodical approach; thus, a comprehensive comparative evaluation of multiple, diverse ML algorithms becomes essential to empirically identify which techniques offer the most effective and resilient predictions for specific BES applications. This type of broad comparative assessment is crucial because different ML algorithms react uniquely to data imperfections such as noise, outliers, and nonlinear relationships. By systematically testing a range of models, rather than relying on a presupposed ‘best’ fit, researchers can uncover algorithms that are optimally suited to the specific characteristics of their data set. The development of accurate ML surrogate models for EM systems has immediate practical implications. Such models could enable real-time process optimization, reduce experimental costs, and accelerate the scale-up of EM technology for industrial biogas upgrading applications.

To our knowledge, no prior study has benchmarked multiple ML models, including advanced deep learning architectures such as one-dimensional CNN (1D-CNN), specifically for predicting biomethane production in microbial electro-methanogenesis systems. This work represents the first comprehensive comparative assessment of ML and deep learning models for EM performance prediction. We evaluate seven diverse algorithms, including neural network-based approaches, a selection of powerful ensemble methods, and an instance-based algorithm. This carefully chosen array of algorithms spans various ML philosophies, ensuring a comprehensive examination of their predictive strengths and weaknesses when applied to EM performance data, with a particular emphasis on biomethane yield. The ultimate objective is to identify the most robust and accurate ML surrogate models from this comparative analysis. Such models are poised to become invaluable tools for guiding data-driven decision-making in the ongoing efforts to design, optimize, and scale up EM systems, thereby contributing to the accelerated adoption of sustainable biomethane as a key component of future renewable energy portfolios.

## 2 Materials and Methods

### 2.1 Experimental Design and Data Sources

Data from two different commercial two chambered, low gap electrochemical reactors were tested during the experimentation (Micro Flow Cell and Electro MP Cell, ElectroCell, Denmark, EM-MFC and EM-EMPC, respectively). Figure 1 presents the EM-MFC experimental setup. Cathodic and anodic chambers were separated by a Nafion N324 membrane. Each system was operated in recirculated batch mode by the addition of a buffer tank in the recirculation line (40 L d^–1^, Watson Marlow, WM205U, 4 channels, USA) of each chamber to increase the working volume to 100 mL. The cathode (carbon felt, thickness 3.18 mm, purity 99.0%, projected surface of 0.001 m^2^, Thermo Scientific, Germany, in adhesion to a stainless steel (SS) collector) and the anode (DSA, ElectroCell, Denmark) were respectively the working (WE) and counter electrode (CE). The EM-EMPC was composed of the same materials, but in an upscaled size: cathode and anode surface were 0.01 m^–2^, and anolyte and catholyte total volumes accounted for 500 mL.

**Figure 1:**
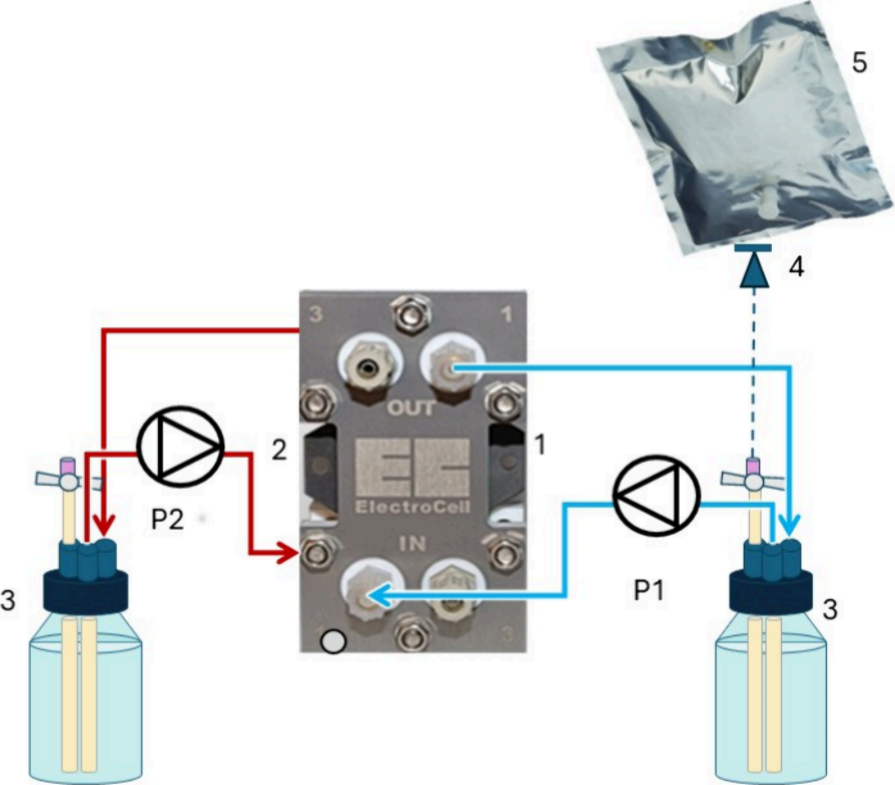
EM-MFC setup. Blue line: cathodic recirculation line. Red line: Anodic recirculation line. 1) Cathode (SS collector); 2) anode (DSA plate); 3) buffer tanks; 4) gas check valve (pressure cut 250 mbar) 5) multifoil gas bag.

The two EMs were operated in a two-electrode configuration, with a potentiostat (Bio-Logic SP50, France) controlling the current provided (galvanostatic mode) and recording the cell voltage and power consumption at different fixed current values (5–50 A m^–2^). Both the anodic and cathodic chambers were filled with methanogenic mineral medium adjusted to a pH of 6.5.^21^ pH was maintained within the range of 6.5–8 and manually corrected when deviations occurred. Cathodic chambers were fed with CO_2_ gas (99.9%, AirLiquide, Spain) as only carbon source, using two different feeding strategies: sampling-feeding or intermittent feeding.

Sampling-feeding strategy was performed by sparging with CO_2_ the buffer tank three times per week after the collection of the samples for 3 min to saturate the medium, leaving an overpressure of 250 ± 20 mbar in the cathodic chamber. Intermittent CO_2_ feeding was performed by providing CO_2_ at atmospheric pressure at different flow rates (140 – 280 mL d^–1^) through the activation of a peristaltic pump (Watson Marlow, WM205U, 4 channels, USA) four times per day for 15 to 30 min each. Liquid phase was renewed weekly as samples were collected (5 mL) and analyzed.

Gas pressure in the reactor’s headspace was measured both before sampling and after feeding (or sampling in the intermittent feeding mode) using a differential manometer (Model-Testo-512; Testo, Germany). For gas analysis, gas samples were collected using a glass syringe (5 mL) before taking liquid samples. These samples were then analyzed using a Micro-GC (Agilent 490 Micro GC system, Agilent Technologies, US). The Micro-GC was equipped with two columns: a CP-molesive 5A column used for analyzing methane (CH_4_), carbon monoxide (CO), hydrogen (H_2_), oxygen (O_2_), and nitrogen (N_2_), and a CP-Poraplot U column used for analyzing carbon dioxide (CO_2_). Both columns were connected to a thermal conductivity detector (TCD). The partial pressure of hydrogen (pH_2_) and CO_2_ (pCO_2_) were calculated by considering the total pressure measured using the differential manometer before gas sampling and the composition of the gas detected in the biocathode’s headspace. The concentrations of dissolved H_2_ and CO_2_ were determined based on Henry’s law at a temperature of 25 °C. Mols of CO_2_ available to be converted were calculated as in conditions of saturation in the sampling feeding routine, while in the intermittent feeding phase they were calculated according to the gas flow-rate operated. Volatile fatty acids (VFAs) and alcohols present in the liquid phase were subjected to analysis using a gas chromatograph (GC) (Agilent 7890A, Agilent Technologies, USA). The GC was equipped with a DB-FFAP column and a flame ionization detector (FID). Acetic acid production was expressed in mg L^–1^ and mol produced point by point, to be compared with methane carbon consumption. Parameters such as electrical conductivity (EC), pH, and optical density (OD_600_) were also measured in the EM reactors liquid samples. All the relevant electric parameters (current [A], voltage [V], power [W] and charge [C]) have been recorded by the potentiostat every 5 min or variation of 0.1 V in the voltage detected by the instrument. The average current density applied has been calculated as the current set normalized by the cathodic surface between each gas sampling point. Table S1 in the Supporting Information summarizes the conditions applied and reactors used for each data set generated.

### 2.2 Model Development and Evaluation Strategy

This study systematically evaluated seven supervised ML algorithms representing three distinct approaches for predicting biomethane production via EM: neural networks, ensemble learning, and instance-based learning. Two neural networks (Multilayer Perceptron (MLP) and 1D-CNN) were selected for their ability to model complex nonlinear relationships. MLP is a feed-forward neural network that maps inputs to outputs through fully connected layers, while 1D-CNN applies convolution filters that detect relationships and patterns between variables, allowing it to learn local feature interactions. Gradient Boosting Regressor (GBR) and Adaptive Boosting (AdaBoost) are tree-based ensemble methods: GBR improves accuracy by iteratively reducing prediction errors from previous trees, and AdaBoost increases the influence of difficult-to-predict samples by adjusting their weight during training. The stacking regressor combines predictions from multiple different models, and a second stacking configuration uses gradient boosting as the meta-model to better capture nonlinear behavior. In contrast, K-Nearest Neighbors (kNN) is a simple instance-based method that makes predictions by comparing new samples to the most similar data points in the training set.^22^ Together, these seven algorithms allow comparison between fundamentally different learning strategies, ranging from simple distance-based reasoning to deep learning architectures capable of modeling complex interactions.

#### 2.2.1 Data Source and Preprocessing

The primary data set **applied** for model training and testing was derived from our own experiments as detailed in Section 2.1. The data set included five input features: optical density at 600 nm (OD_600_), pH, EC (mS/cm), average applied current (mA), and CO_2_ availability (mol). These features were selected based on their established influence on methanogenic activity and their ease of measurement in industrial settings. The target output variable for prediction was biomethane yield. Data quality was ensured by including only samples where all parameters were measured without sensor malfunctions or experimental anomalies. Following this criterion, the final data set composed of 133 experimental samples derived from the EM reactors (11 independent reactor batches as described in Table S1) operated under a sampling-feeding CO_2_ strategy across a full current range (5–50 A m^–2^). In each batch, multiple sampling events were conducted to measure OD_6_00, pH, EC, applied current, and CO_2_ availability, together with the resulting biomethane production. No missing values were present in the final data set, and outlier analysis using the interquartile range method confirmed all measurements were within expected experimental bounds.

Prior to model training, the input features were standardized using the MinMaxScaler technique, scaling each feature to a range between 0 and 1. This min-max normalization was chosen over z-score standardization to preserve the bounded nature of our features and improve convergence for neural network models. This normalization step is crucial for algorithms sensitive to feature magnitudes, ensuring consistent data representation and preventing features with larger scales from dominating the learning process. An exception was made for the kNN algorithm, which does not inherently require feature normalization for its distance-based calculations.

#### 2.2.2 Model Training, Hyperparameter Optimization, and Validation

The data set was randomly partitioned into a training set (90% of the samples) and a testing set (10% of the samples). The test set was kept strictly unseen during model training and hyperparameter optimization to ensure that the reported performance reflects true generalization. To minimize bias arising from a single random split, particularly given the modest data set size, this partitioning process was repeated 30 times. In each iteration, the data were reshuffled and repartitioned without replacement, preserving the 90/10 proportion, and the model was trained from scratch. After 30 repetitions, the mean and standard deviation of the evaluation metrics were calculated (detailed in Section 2.3), providing a more reliable assessment of predictive performance and stability across different train–test configurations. A schematic of this modeling workflow, from data preprocessing through to evaluation, is presented in Figure 2.

**Figure 2:**
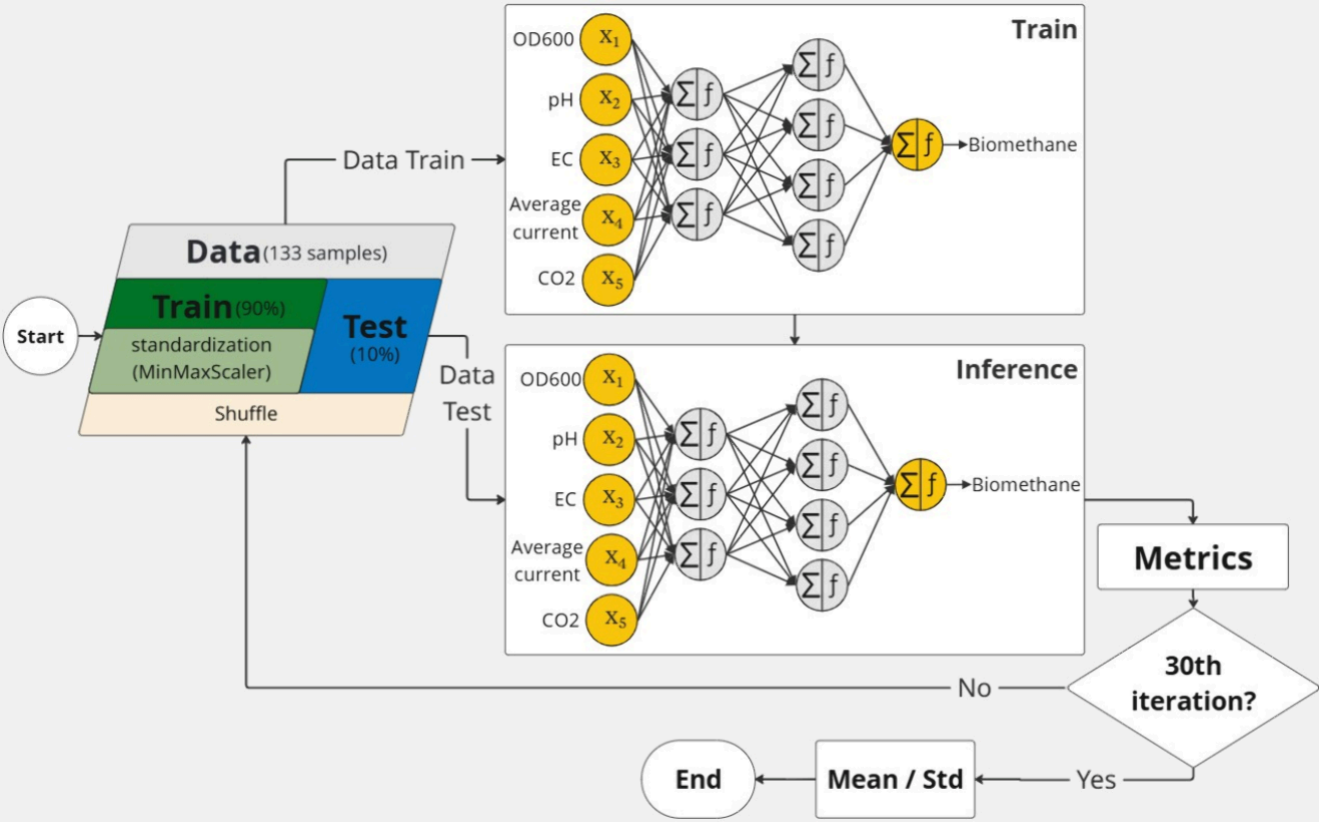
Flowchart of the modeling approach, from data preprocessing, training, testing, evaluation and final assessment.

For the MLP and CNN models, hyperparameter optimization was performed using the KerasTuner library^23^ to identify the optimal architectural configurations. As detailed in Table S2, a predefined search space for hyperparameters, including the number of layers, neurons per layer, activation functions, dropout rates, learning rates, and optimizers, was explored with the objective function of minimizing the MSE from the best individual execution. The configuration yielding the minimal validation mean squared error (MSE) was selected for the final model training. The specific hyperparameters for all seven models are presented in Table S3, with the optimized architectures for CNN and MLP depicted in Figures 3 and 4, respectively. The configuration yielding the minimal validation mean squared error (MSE) was selected for the final model training. The specific hyperparameters for all seven models are presented in Table S3, with the optimized architectures for CNN and MLP depicted in Figures 3 and 4, respectively.

**Figure 3:**
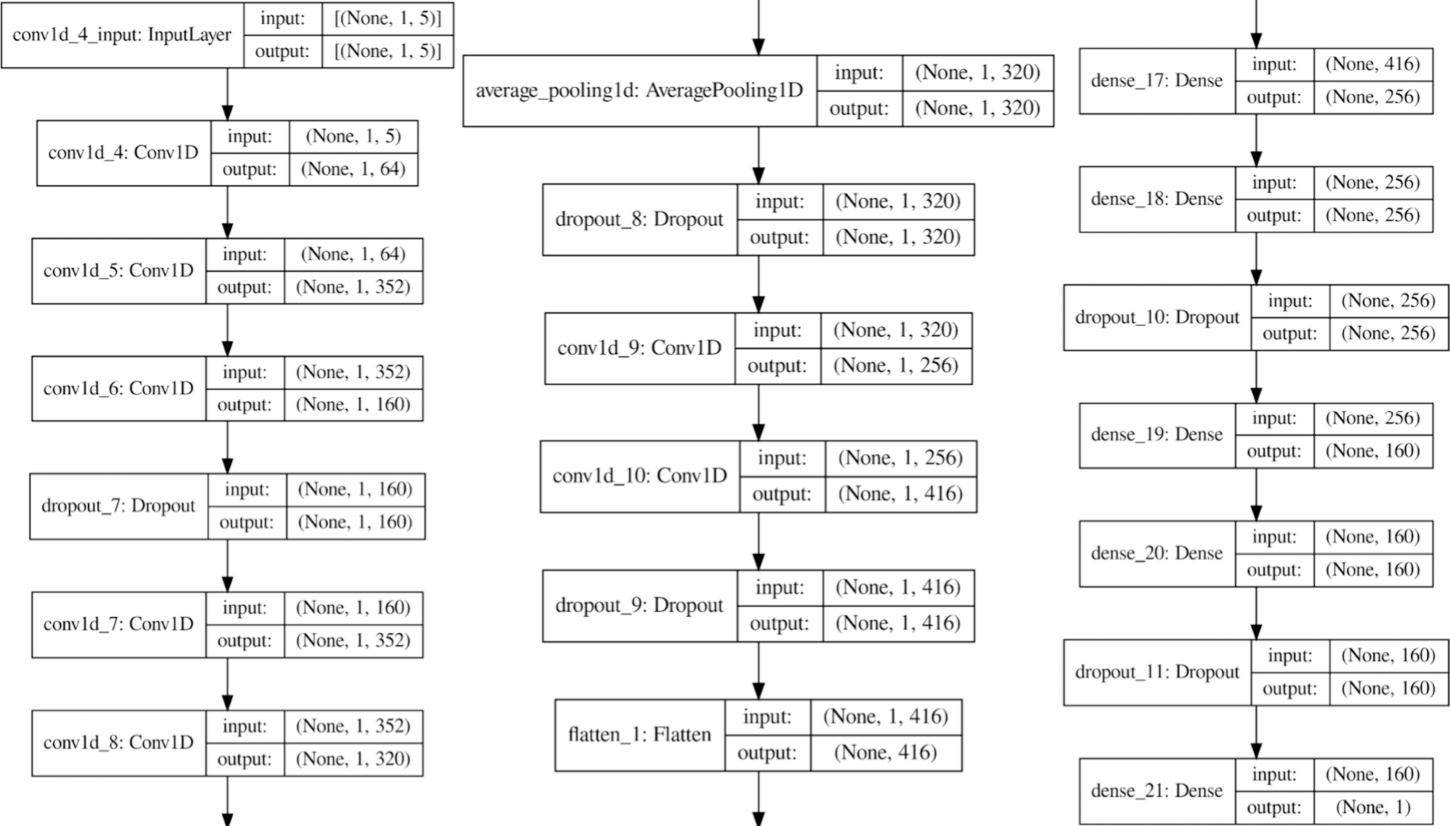
CNN model architecture.

**Figure 4:**
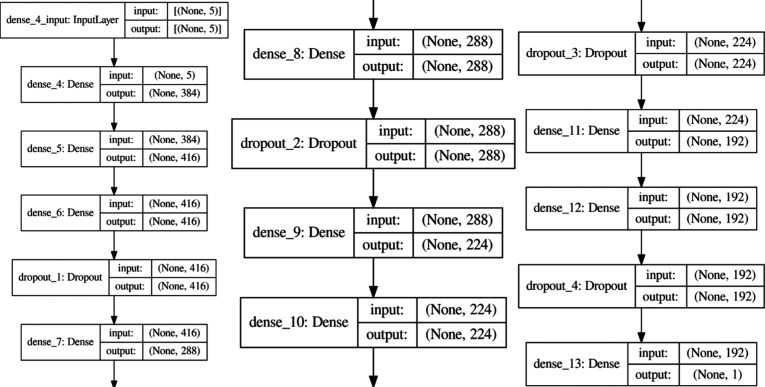
MLP model architecture.

The computational cost of each technique was also assessed by measuring the average training time and the average inference time per sample on a computer equipped with 40 physical cores and 196 GB of memory.

#### 2.2.3 Software and Libraries

All model development and data analysis were conducted using the Python programming language. Neural networks (MLP and CNN) were implemented using the Keras library with TensorFlow^24^ as the backend. Ensemble methods and kNN were implemented primarily using the Scikit-learn (Sklearn) library,^25^ with Mlxtend employed for the stacking regressors.^26^

### 2.3 Model Performance Evaluation Metrics

The predictive performance of the developed ML models was rigorously evaluated using a comprehensive set of standard regression metrics. These metrics were calculated for each of the 30 computational runs (as described in Section 2.2.2), and the final reported performance for each model consists of the mean and standard deviation of these metrics, offering insights into both accuracy and stability. The selected metrics are detailed below:

- Pearson R: The Pearson correlation coefficient is calculated to measure the statistical relationship between two variables. The resulting values range from −1 to +1, where +1 indicates the strongest possible agreement and −1 represents the strongest possible disagreement. This metric is computed using eq 2.1.
r=∑i=1n(Yi−Ý)(Ŷi−y^´)∑i=1n(Yi−Ý)2∑i=1n(Ŷi−y^´)2 2.1
  where *Ŷ* is the vector of predicted values,, *Y* is the vector of actual values, Ý and y^´ ^́ are the mean of the actual and predicted values, respectively, n is the number of data points.
- Spearman R: This coefficient is a nonparametric measure of the monotonicity of the relationship between two data sets. Like other correlation coefficients, its values range from −1 to +1, with 0 indicating no correlation. A correlation of −1 or +1 represents an exact monotonic relationship. Positive correlations indicate that as x increases, y also increases, while negative correlations indicate that as x increases, y decreases. This metric is computed using eq 2.2.
$$\rho =1-\frac{6\sum (Y_{i}-{Ŷ}_{i}{)}^{2}}{n(n^{2}-1)}$$ 2.2
- Factor of 2 (Fac2): The percentage of predicted values whose ratio with the actual values falls between 0.5 and 2, indicating a factor-of-2 agreement, is calculated. Its values range from 0 to 1, and the closer to 1 the better. This metric is computed using eq 2.3.
$$Fac2=\frac{1}{n}\sum_{i=1}^{n}1\left(0.5\leq \frac{{ŷ}_{i}}{y_{i}}\leq 2\right)$$ 2.3
  where 1(·) is an indicator function that returns 1 if the condition inside is true and 0 otherwise.
- Mean Absolute Error (MAE): The mean absolute difference between the predicted and actual values is calculated. Its values range from 0 to + ∞, and the closer to 0 the better. This metric is defined by eq 2.4.
$$MAE=\frac{1}{n}\sum_{i=1}^{n}|Y_{i}-{Ŷ}_{i}|$$ 2.4
- Mean Squared Error (MSE): The mean squared difference between the predicted and actual values is calculated. Its values range from 0 to + ∞, and the closer to 0 the better. This metric is defined by eq 2.5.
$$MSE=\frac{1}{n}\sum_{i=1}^{n}(Y_{i}-{Ŷ}_{i}{)}^{2}$$ 2.5
- Normalized Mean Squared Error (NMSE): The normalized mean squared difference between the predicted and actual values is calculated. Its values range from 0 to + ∞, the closer to 1 the better and the smaller it is than 1 it means that the mean squared error does not vary more than 1 variance of the observed series. This metric is defined by eq 2.6.
$$NMSE=\frac{MSE}{\sigma {\left(Y\right)}^{2}}$$ 2.6
  where σ is the standard deviation.
- Root Mean Squared Error (RMSE): The root mean squared difference between the predicted and actual values is calculated. Its values range from 0 to + ∞, and the closer to 0 the better. This metric is defined by eq 2.7.
$$RMSE=\sqrt{\frac{1}{n}\sum_{i=1}^{n}(Y_{i}-{Ŷ}_{i}{)}^{2}}$$ 2.7
- Normalized Root Mean Squared Error (NRMSE): The normalized root mean squared difference between the predicted and actual values is calculated. Its values range from 0 to + ∞, the closer to 1 the better and the smaller it is than 1 it means that the root mean squared error does not vary more than 1 standard deviation from the observed series. This metric is defined by eq 2.8.
$$NRMSE=\frac{RMSE}{\sigma \left(Y\right)}$$ 2.8

## 3 Results and Discussion

This section presents a comprehensive analysis of the predictive performance of various ML algorithms for estimating biomethane production rates from EM. The evaluation is based on standard regression metrics, including R,^2^ PearsonR, SpearmanR, Fac2, MAE, MSE, NMSE, RMSE, and NRMSE.

### 3.1 Comparative Analysis of Model Performance

A comprehensive summary of the key regression metrics, including the mean and standard deviation averaged over 30 randomized train-test splits, is presented in Tables 1a and b. The average training and inference times are provided in Table 2, and the prediction error plots for each model are shown in Figure 5.

**Table 1:** (a) Mean and (b) Standard Deviation Values of the Performance Metrics Obtained for Each Model Computed across the 30 Experiments^a^

**(a) Mean values of the metrics obtained for each model**									
**Type**	**R** ^**2**^	**PearsonR**	**SpearmanR**	**Fac2**	**MAE**	**MSE**	**NMSE**	**RMSE**	**NRMSE**
AdaBoost	0.81	0.601	0.584	0.68	16.73	464.85	0.326	21.23	0.562
CNN	**0.934**	**0.835**	**0.824**	**0.83**	**9.893**	**206.11**	**0.145**	**13.29**	**0.352**
Gradient Boosting	0.693	-	-	0.59	21.47	702.27	0.493	26.09	0.691
KNN	0.815	0.63	0.604	0.73	16.72	430.06	0.302	20.37	0.54
MLP	0.693	-	-	0.6	21.42	700.44	0.491	26.06	0.69
Stacking Regressor	0.819	0.591	0.586	0.67	17.46	450.19	0.316	20.91	0.554
Stacking Regressor with Gradient Boosting	0.818	0.597	0.568	0.68	17.75	453.89	0.318	21.03	0.557

**(b) Standard deviation of the metrics obtained for each model**								
**Type**	**PearsonR**	**SpearmanR**	**Fac2**	**MAE**	**MSE**	**NMSE**	**RMSE**	**NRMSE**
AdaBoost	0.141	0.136	0.15	3.36	169.98	0.119	3.821	0.101
CNN	0.142	0.126	0.12	4.5	159.83	0.112	5.523	0.146
Gradient Boosting	-	-	0.14	4.48	236.75	0.166	4.728	0.125
KNN	0.138	0.14	0.1	3.7	167.83	0.118	3.945	0.104
MLP	-	-	0.13	4.47	235.86	0.165	4.686	0.124
Stacking Regressor	0.173	0.194	0.12	3.42	154.39	0.108	3.662	0.097
Stacking Regressor with Gradient Boosting	0.156	0.176	0.14	3.22	144.9	0.102	3.444	0.091

a The best-performing results are highlighted in bold.

**Table 2:** Average Training Time for Each Model and the Average Inference Time Per Sample Are Measured^a^

**Type**	**Training Time (seconds)**	**Inference Time per Sample (milliseconds)**
AdaBoost	0.521	3.517
CNN	7.482	6.666
Gradient Boosting	4.071	0.967
kNN	**0.002**	**0.273**
MLP	7.349	6.243
Stacking Regressor	0.144	0.412
Stacking Regressor with Gradient Boosting	8.723	1.357

a The best-performing results are highlighted in bold.

**Figure 5:**
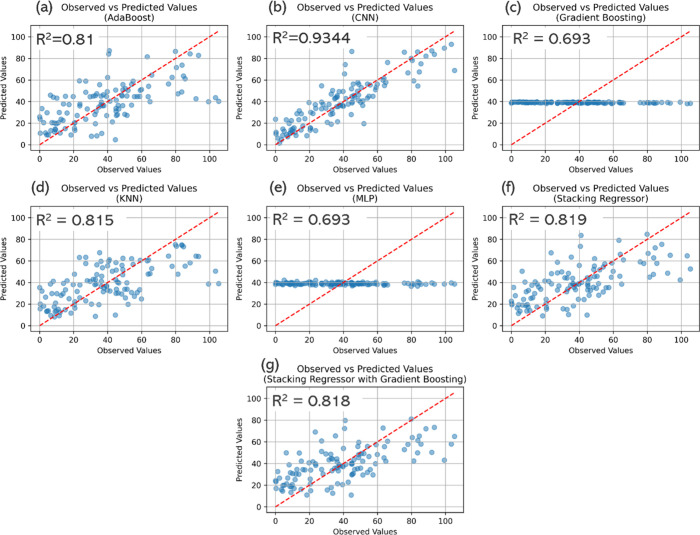
Performance of (a) Adaboost, (b) CNN, (c) Gradient boosting, (d) kNN, (e) MLP, (f) Stacking regressor, and (g) stacking regressor with gradient boosting, in predicting biomethane concentration.

The results presented in Tables 1 and 2, and Figure 5, clearly establish a performance hierarchy among the tested algorithms. The 1D-CNN consistently outperformed all other models across every key metric. It achieved the highest R^2^ value of 0.934, the strongest correlation coefficients (PearsonR of 0.835 and SpearmanR of 0.824), and the best Fac2 score of 0.831, indicating that over 83% of its predictions fell within a factor of 2 of the experimental values. Furthermore, the CNN produced the lowest prediction errors (MAE = 9.9, MSE = 206.1, and RMSE = 13.3), confirming its superior accuracy and ability to minimize large deviations. This top-tier performance was also highly stable, as shown by the low standard deviations in Table 1b.

A cluster of models, AdaBoost, kNN, Stacking Regressor, and Stacking Regressor with Gradient Boosting, showed moderate but comparable performance. Their R^2^ values hovered around 0.8, while PearsonR and SpearmanR values were around 0.60, and Fac2 ranged between 0.67–0.73. Their MAE and MSE were higher (MAE ∼ 16–17, MSE ∼ 430–465), indicating less precise predictions and a greater tendency to deviate from experimental outcomes. However, these models still performed better than the weakest algorithms, reflecting reasonable robustness but ultimately limited by their inability to fully capture the complex, nonlinear dependencies inherent in EM process data.

Finally, the MLP and Gradient Boosting models recorded the poorest performance. Their Fac2 scores were the lowest (below 0.60), and their error metrics were nearly three times worse than the CNN. These two models also exhibited the greatest variability across the 30 runs, as evidenced by their high standard deviations (Table 1b). This points to model instability and a tendency to overfit or underfit, a common challenge when applying some algorithms to limited and intrinsically variable data sets like those from EM systems. Furthermore, the inability to compute certain metrics, shown as hyphens in Table 1, was attributable to the fact that predictions remained constant across all inputs, as confirmed by the performance graphs in Figure 5c,e.

While predictive accuracy is paramount, computational speed is also critical for real-world deployment. As shown in Table 2, kNN was the fastest algorithm in both training (0.002 s) and inference (0.27 ms/sample). However, its inferior accuracy reinforces that computational speed alone does not compensate for a loss in predictive power. In contrast, the CNN, despite being one of the slower models to train (7.48 s), remained highly practical for real-time use, predicting each new sample in approximately 6.7 ms. This is orders of magnitude faster than typical first-principles simulators for BESs,^27^ giving the CNN a rare blend of accuracy, reliability, and deployment speed.

The superior performance of the 1D-CNN can be explained by the structured interactions between the operational parameters that govern EM. Although the input is a five-feature vector rather than a temporal sequence, the features are not independent; pH influences microbial growth (OD_6_00), conductivity affects electron transport, and applied current is linked to CO_2_ availability through cathodic electrochemical reactions. These physicochemical dependencies form meaningful local relationships within the feature vector. The convolutional filters in the 1D-CNN learn these relationships automatically by detecting spatially local patterns such as [pH–OD_6_00] or [current–CO_2_ availability] interactions. In contrast, an MLP treats all features globally and assigns weight to each feature independently, making it less efficient at capturing synergistic effects unless large data sets are available. Gradient boosting and AdaBoost rely on recursive feature splitting and are therefore strong for monotonic or tree-like relationships, but they struggle to model combined, nonlinear electro-biological interactions between multiple variables. The 1D-CNN learns hierarchical, multivariate feature combinations through convolution and weight sharing,^28^ enabling it to detect how two or more operational factors jointly influence methane production. This architectural advantage explains why the 1D-CNN outperformed the MLP and traditional ensemble models, despite the small data set.

To further assess the generalization capability of the 1D-CNN model and address potential overfitting concerns, the model’s performance was compared across the training and testing subsets. As presented in Table 3, the R^2^ and RMSE values for the training set were 0.955 and 8.99, respectively, while for the testing set, these values decreased to 0.875 and 15.21. Although this indicates a degree of overfitting, expected given the data set size and model complexity, the difference between training and testing performance remains moderate. Importantly, the test-set R^2^ remains high, demonstrating that the 1D-CNN was able to generalize to unseen data while capturing nonlinear interactions relevant to EM performance.

**Table 3:** Comparison of 1D-CNN Performance on Training and Testing Datasets (Mean R^2^ and RMSE over 30 Iterations)

**Data set**	**Mean R** ^**2**^	**Mean RMSE**
Training Set	0.955	8.99
Testing Set	0.875	15.21

To date, only a handful of studies have directly applied ML to model methane production in EM or related MEC systems. One of the most relevant studies is by Xiao et al. (2021),^29^ who applied a two-stage NARX-BP hybrid neural network to predict methane yields in MEC biocathodes. Their model achieved an R^2^ of 0.918 and a mean squared error of 0.0652, demonstrating excellent predictive performance and the value of neural architectures that can handle feedback and temporal dependencies in BESs. Remarkably, our CNN model not only matches but slightly outperforms this established benchmark (R^2^ = 0.934 vs 0.918). This is significant given that the NARX approach is specifically tailored for time series data and incorporates feedback, while in our case, the CNN approach relies on a fixed set of physicochemical and operational variables at each measurement point. The strong performance of CNN model suggests that spatial or local feature extraction, as enabled by convolutional architectures, can be highly effective in mapping complex relationships in EM data, even in the absence of detailed temporal inputs.

Beyond methane, ML approaches have been applied to other BES outputs. For instance, Li et al. (2024)^19^ reported XGBoost models achieving R^2^ > 0.90 for acetate and ethanol production in MES, illustrating that ensemble methods can handle multistep, nonlinear biochemical pathways. However, as our results show, deep neural models like CNN can outperform these ensemble approaches, even on a moderately sized data set, especially as system complexity and interaction between variables increase. This observation echoes findings from recent reviews^30^ emphasizing the growing potential of neural networks and hybrid deep learning models for modeling complex, nonlinear BES processes. For example, Yoon et al. (2024)^18^ noted that RF models performed markedly better when trained on MECs with a single substrate compared to mixed-substrate data. Also, our input feature set, while comprehensive, does not encode certain potentially influential factors, such as specific electrode surface chemistry, microbial community structure, or trace micronutrient concentrations, that can strongly influence EM performance. Yoon et al. (2024)^18^ specifically noted that the inability to numerically represent and include certain qualitative features was a limiting factor in their work as well.

### 3.2 Analysis of Feature Contributions Using SHAP

To move beyond predictive accuracy and gain mechanistic insights into the factors governing biomethane production in EM systems, we conducted a feature importance analysis using the best-performing CNN model. The SHAP (SHapley Additive exPlanations) technique was employed to provide robust and interpretable insights into how each input feature influenced the model’s predictions, both globally across the entire data set and locally for individual samples. The global feature importance, calculated as the mean absolute SHAP value for each feature, provides a clear ranking of the factors driving biomethane production predictions (Figure 6).

**Figure 6:**
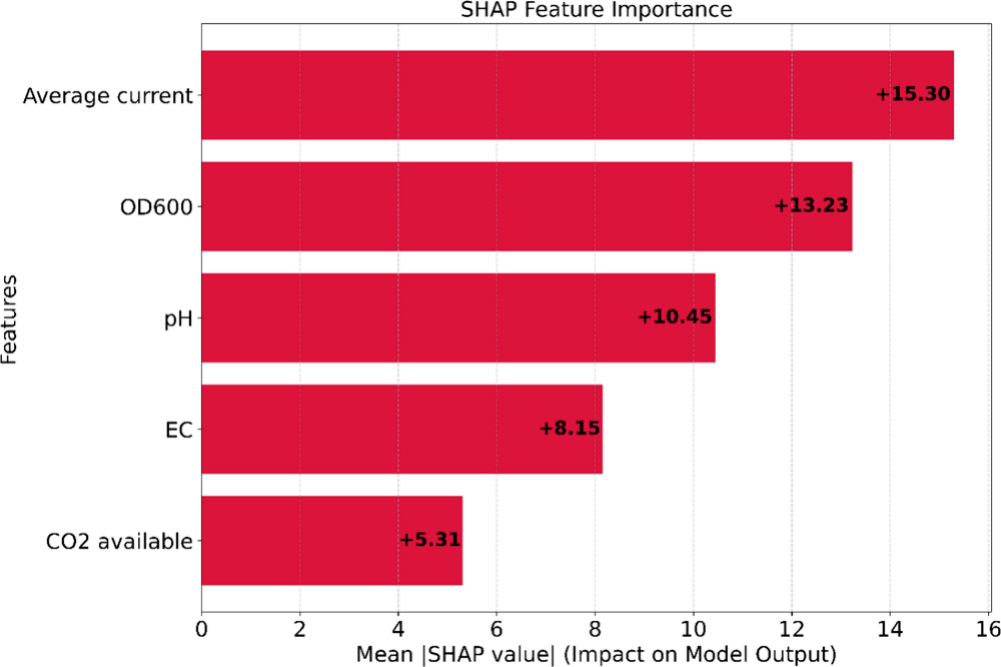
SHAP Feature Importance using whole data

The analysis identified average current as the most influential feature, followed closely by optical density (OD_6_00) and pH. This ranking aligns well with the fundamental principles of bioelectrochemical methanogenesis.^31^ The dominance of average current as the primary predictor is consistent with the electron-driven nature of EM. Current directly governs the rate of electron supply to the biocathode, which serves as the primary energy source for electromethanogenic microorganisms. Higher current densities provide more electrons for CO_2_ reduction to methane, either through direct electron transfer or via electrochemically produced H_2_ as an intermediate. This finding corroborates previous studies that identified current density as a critical operational parameter in bioelectrochemical systems.^8^ Notably, this observation extends beyond EM systems; Li et al. (2024)^19^ similarly identified current as one of the primary influencers in MES systems for both acetate and ethanol production, emphasizing that electron availability directly influences microbial electron utilization for inorganic carbon fixation.

The high importance of OD_6_00, a proxy for microbial biomass concentration, underscores the biological nature of the process. Greater microbial density typically correlates with enhanced biocatalytic capacity in EM, provided other conditions remain favorable. This parameter’s significance suggests that biofilm development and microbial growth kinetics play crucial roles in determining system performance, consistent with observations in related MES systems.^32,33^

The SHAP summary plot (Figure 7) provides deeper insights into how individual feature values influence predictions across the data set. As expected, high current values consistently showed positive SHAP contributions, indicating a strong positive correlation with biomethane production. This monotonic relationship supports the electron-limitation hypothesis in many EM systems, where methane production increases with electron availability until other factors become limiting. The SHAP dependence for average current also exhibits a clear “knee.” By analyzing the exported SHAP (current) values (Figure 7) and back-transforming the normalized current axis to physical units using the min–max range applied in preprocessing (3–50 A m^–2^; Section 2.2.1; Table S1), we find that the SHAP contribution of current becomes positive at ∼ **17.1 A m** ^**–2**^ and increases rapidly up to ∼ **17.5–18.0 A m** ^**–2**^, where the marginal gain drops below ∼ 5–20% of its peak and remains low thereafter (plateau). The SHAP contribution continues to grow only slowly, reaching ∼ 75% of its maximum by ∼ **45 A m** ^**–2**^. This behavior accords with established electromethanogenic constraints: at low–moderate currents, methane formation is electron-limited, whereas beyond ∼ **18 A m** ^**–2**^ the system transitions to regimes dominated by CO_2_/proton mass-transfer limitations at the cathode, increased competition from the hydrogen evolution reaction at higher overpotentials, and finite cathodic biofilm electron-uptake capacity, all of which diminish the incremental benefit of further current increases.^8,34,35^ Similar to current, elevated OD_6_00 values predominantly exhibited positive SHAP values, confirming that higher microbial densities generally enhance methane production. However, the relationship appears to plateau at very high biomass concentrations, possibly due to mass transfer limitations or substrate depletion in dense biofilms.

**Figure 7:**
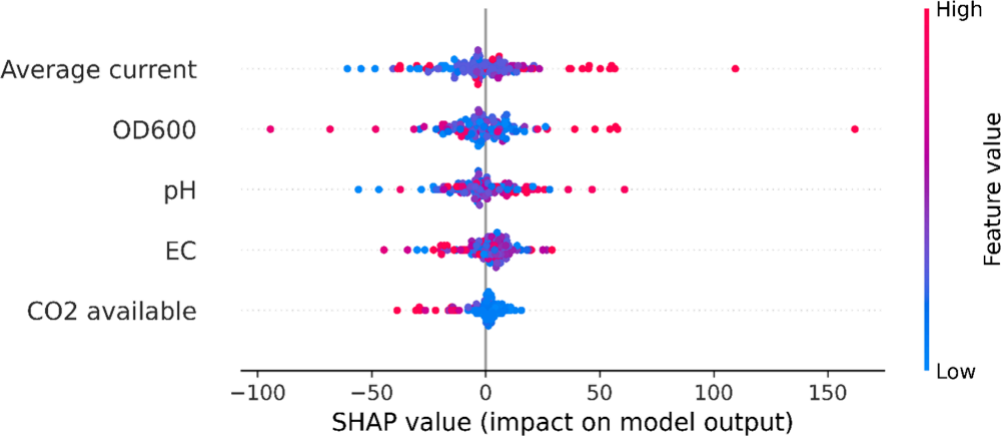
SHAP summary using whole data. Each point represents a single observation. The horizontal position indicates the SHAP value (impact on methane prediction), while the color gradient from blue (low feature value) to red (high feature value) shows the magnitude of the input feature associated with each point.

The model captured pH as a significant factor, reflecting its critical role in maintaining optimal conditions for methanogenic archaea. In addition, the SHAP dependence plot for pH reveals a distinct nonlinear trend. The model identifies that pH values between ∼ 6.6 and 8.0 result in positive SHAP contributions to methane production, with the most pronounced positive influence centered around ∼ 6.6–7.6. Outside this window, SHAP values become negative, indicating reduced methane formation. This behavior aligns with established methanogenic physiology, where methane generation follows a characteristic parabolic rate profile centered near neutral pH. Under acidic or alkaline stress, methanogenic activity declines sharply due to proton transport imbalance, disruption of intracellular redox potential, and inhibition or denaturation of key metabolic enzymes.^36,37^ Importantly, the CNN was not supplied with kinetic equations or prior knowledge of methanogen physiology; instead, it learned this behavior solely from experimental data, demonstrating that interpretable machine learning can capture canonical biological kinetics without requiring explicit mechanistic models.

Interestingly, EC showed a complex, nonmonotonic relationship with biomethane production. Both high and low EC values could contribute positively or negatively depending on other conditions. This complexity likely reflects EC’s dual role: while adequate ionic strength is necessary for maintaining osmotic balance and facilitating charge transfer, excessive conductivity may indicate salt stress or inhibitory conditions. Surprisingly, higher CO_2_ availability consistently showed negative SHAP values. This counterintuitive finding warrants careful interpretation. One possibility is that excessive CO_2_ leads to acidification of the cathode microenvironment, inhibiting methanogenic activity. Alternatively, in our experimental setup, high CO_2_ partial pressures may have been associated with mass transfer limitations or suboptimal gas–liquid equilibria.

To understand the model’s behavior in specific instances, we performed local SHAP analysis on seven representative samples: five where predictions closely matched observations and two outliers where the model’s predictions diverged significantly (Figure 8).

**Figure 8:**
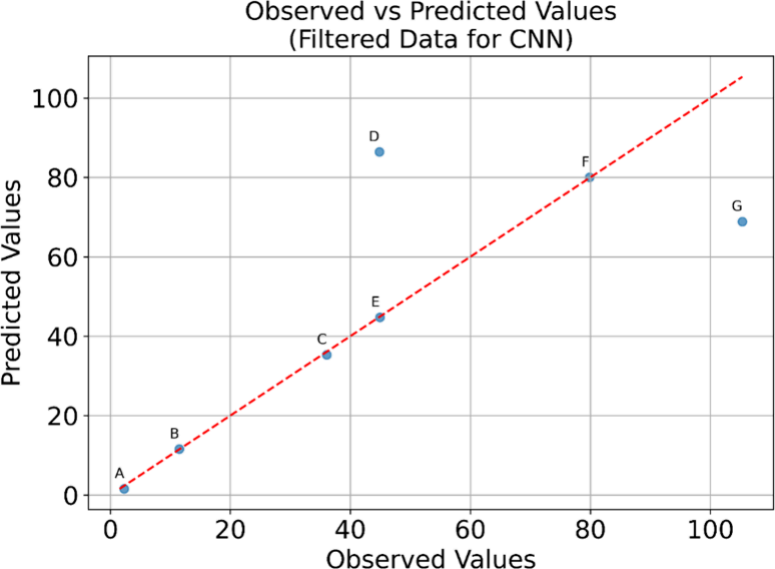
Zoom-in of performance of CNN in predicting biomethane concentration.

The force plots for individual samples A-G were also plotted in Figure 9 and revealed instance-specific feature interactions:

1. **For low biomethane production scenarios** (Samples A-C), EC and CO_2_ availability acted to increase predictions, potentially compensating for limitations in current or biomass. This suggests the model learned that under electron-limited conditions, higher ionic strength might facilitate better electron transfer efficiency.
2. **For high biomethane production scenarios** (Samples E and F), EC and CO_2_ showed negative contributions, preventing overestimation. This indicates the model captured inhibitory effects that become prominent only at high production rates.
3. **In samples with prediction errors** (Samples D and G), conflicting feature contributions were observed. For instance, when OD_6_00 and current both strongly pushed predictions downward despite moderate observed production, this suggests potential noncaptured factors such as microbial community shifts or temporal dynamics not encoded in our steady-state features, such as increased acetoclastic metanogenesis (point D) corresponding to an increased availability of acetic acid or their inhibition (point G).

**Figure 9:**
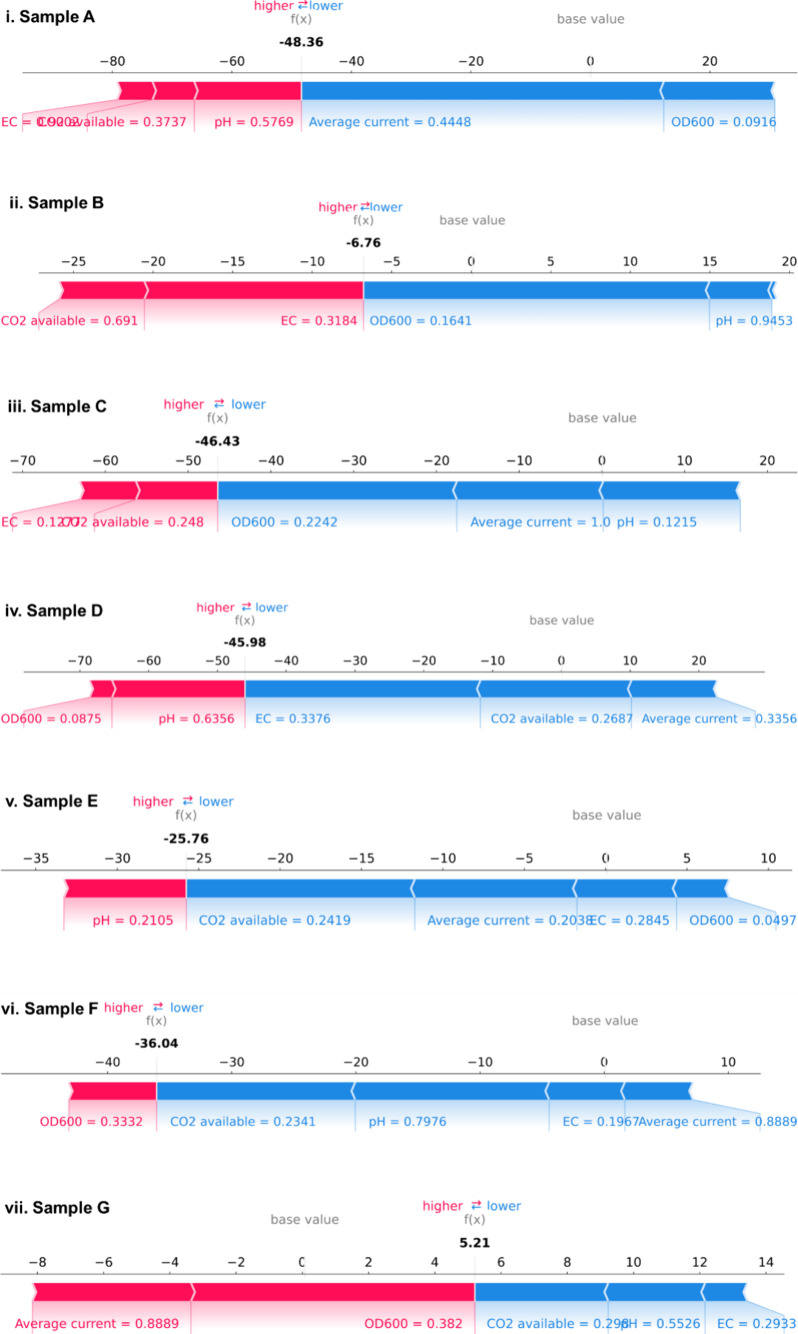
SHAP force plots (i - vii) for samples A-G, as presented in Figure 8.

These findings demonstrate that while EM is fundamentally an electrochemical process (hence current’s dominance), its optimization requires careful orchestration of biological (OD_6_00), chemical (pH, EC), and mass transfer (CO_2_) parameters. The successful capture of these multifaceted interactions by the CNN model validates its utility as a tool for process understanding beyond mere prediction.

From a practical standpoint, this work delivers an efficient surrogate model capable of real-time predictions (6.7 ms per sample) that could replace computationally expensive mechanistic models for process control and optimization. The identified feature importance hierarchy suggests prioritizing current density control and biomass retention strategies while carefully managing pH and CO_2_ feeding to avoid inhibitory conditions.

However, several limitations should be acknowledged. We recognize that the data set used in our analysis (N = 133) is small for training deep learning models and while the CNN model performed best under the given conditions, we view the model as an initial proof-of-concept rather than a fully generalizable deep learning framework. Also, the model was trained on data from a specific reactor configuration with limited operational diversity. The absence of temporal dynamics, microbial community data, and electrode-specific characteristics in the feature set likely constrains the model’s ability to capture all relevant phenomena. The surprising negative influence of CO_2_ availability particularly highlights potential gaps in our experimental design or unconsidered interaction effects.

Future research should focus on integrating additional data such as microbial community composition, electrode material properties, or detailed time-series measurements could capture deeper biological and electrochemical dynamics. Developing more generalizable models will benefit from larger and more diverse data sets, which could be achieved through standardized data-sharing initiatives or multilaboratory collaborations. Integration of temporal dynamics through recurrent architectures or physics-informed neural networks could further enhance predictive capabilities. Additionally, investigating the unexpected CO_2_-methane relationship through targeted experiments could yield valuable insights for process optimization.

## 4 Conclusion

This study provides a comparative evaluation of seven machine learning algorithms for predicting biomethane production in microbial electro-methanogenesis (EM) systems. Among the models tested, the one-dimensional CNN consistently delivered the highest accuracy (R^2^ = 0.934, MAE = 9.89, RMSE = 13.29) and generalization ability, with over 83% of predictions falling within a factor of 2 of experimental data. Its strong performance, coupled with rapid inference times, highlights CNN’s suitability as a practical surrogate model for real-time EM process optimization.

Beyond predictive capability, SHAP-based interpretability analysis identified average current as the dominant driver of methane production, followed by microbial density (OD_6_00) and pH. These results reinforce the electron-driven nature of EM while underscoring the importance of biological and chemical conditions. The analysis also revealed more complex interactions, such as the nonmonotonic role of electrical conductivity and the counterintuitive negative influence of high CO_2_ availability, pointing to areas that warrant further experimental investigation.

The ability of the CNN model to capture both global feature importance and local, context-specific interactions demonstrates its value not only as a predictive tool but also as a means of deepening mechanistic understanding of EM. While the present study was limited by data set size, reactor configuration, and the absence of temporal and microbial community data, it establishes a clear framework for applying advanced ML to EM systems. Future work should extend these models with broader data sets, physics-informed architectures, and additional biological and material descriptors to further enhance their robustness and generalizability.

## Supplementary Material



## Notes

Tables describing the different reactors used in generating the experimental data sets, hyperparameter range used in KerasTunner to fine-tune the MLP and CNN models, and hyperparameters configured for each model (PDF)

## References

[1] Rasapoor M., Young B., Brar R., Sarmah A., Zhuang W. Q., Baroutian S. (2020). Recognizing the challenges of anaerobic digestion:
Critical steps toward improving biogas generation. Fuel..

[2] Servin-Balderas I., Wetser K., ter Heijne A., Buisman C., Hamelers B. (2024). CO_2_-based methane: an
overlooked solution for the energy transition. Energ Sustain Soc..

[3] Carrillo-Peña D., Escapa A., Hijosa-Valsero M., Paniagua-García A. I., Díez-Antolínez R., Mateos R. (2024). Bioelectrochemical
enhancement of methane production from exhausted vine shoot fermentation
broth by integration of MEC with anaerobic digestion. Biomass Conversion and Biorefinery.

[4] Dessì P., Rovira-Alsina L., Sánchez C., Dinesh G. K., Tong W., Chatterjee P., Tedesco M., Farràs P., Hamelers H. M. V., Puig S. (2021). Microbial
electrosynthesis: Towards
sustainable biorefineries for production of green chemicals from CO_2_ emissions. Biotechnol. Adv..

[5] Blasco-Gómez R., Ramió-Pujol S., Bañeras L., Colprim J., Balaguer M. D., Puig S. (2019). Unravelling
the factors that influence the bio-electrorecycling of
carbon dioxide towards biofuels. Green Chem..

[6] Batlle-Vilanova P., Rovira-Alsina L., Puig S., Balaguer M. D., Icaran P., Monsalvo V. M., Rogalla F., Colprim J. (2019). Biogas upgrading, CO_2_ valorisation
and economic revaluation of bioelectrochemical
systems through anodic chlorine production in the framework of wastewater
treatment plants. Science of The Total Environment.

[7] Pelaz G., Carrillo-Peña D., Morán A., Escapa A. (2024). Microbial electromethanogenesis
for energy storage: Influence of acidic pH on process performance. J. Energy Storage.

[8] Molognoni D., Bosch-Jimenez P., Rodríguez-Alegre R., Marí-Espinosa A., Licon E., Gallego J., Lladó S., Borràs E., Della Pirriera M. (2020). How operational parameters affect
electromethanogenesis in a bioelectrochemical power-to-gas prototype. Frontiers in Energy Research.

[9] Cai W., Cui K., Liu Z., Jin X., Chen Q., Guo K., Wang Y. (2022). An electrolytic-hydrogen-fed moving bed biofilm reactor
for efficient
microbial electrosynthesis of methane from CO_2_. Chem. Eng. J..

[10] Brandão
Lavender M., Steller J., Liu D., de Rink R., Tofik S., ter Heijne A. (2025). Designing, building and operating
an up-scaled methane producing bioelectrochemical system for power-to-methane. J. Power Sources.

[11] Li Z., Fu Q., Su H., Yang W., Chen H., Zhang B., Hua L., Xu Q. (2022). Model development of bioelectrochemical systems: A
critical review from the perspective of physiochemical principles
and mathematical methods. Water Res..

[12] Tanguay-Rioux F., Nwanebu E., Thadani M., Tartakovsky B. (2023). On-line current
control for continuous conversion of CO2 to CH4 in a microbial electrosynthesis
cell. Biochemical Engineering Journal.

[13] Gadkari, S. Application of artificial intelligence methods for the optimization and control of bioelectrochemical systems. In Scaling Up of Microbial Electrochemical Systems; Elsevier, 2022 pp 437–455. 10.1016/B978-0-323-90765-1.00023-X.

[14] Gharbi R., Gomez Vidales A., Omanovic S., Tartakovsky B. (2022). Mathematical
model of a microbial electrosynthesis cell for the conversion of carbon
dioxide into methane and acetate. J. CO2 Util..

[15] Li C., Guo D., Dang Y., Sun D., Li P. (2023). Application of artificial
intelligence-based methods in bioelectrochemical systems: Recent progress
and future perspectives. Journal of Environmental
Management.

[16] Cai W., Lesnik K. L., Wade M. J., Heidrich E. S., Wang Y., Liu H. (2019). Incorporating
microbial community data with machine learning techniques
to predict feed substrates in microbial fuel cells. Biosens. Bioelectron..

[17] Hess-Dunlop A., Kakani H., Taylor S., Louie D., Eshraghian J., Josephson C. (2025). Time-series forecasting of microbial
fuel cell energy
generation using deep learning. Front. Comput.
Sci..

[18] Yoon S. (2024). Machine learning prediction
of hydrogen production and current density
in microbial electrolysis cells. Bioresour.
Technol..

[19] Li C., Li H., Li P., Dang Y., Sun D., Guo D. (2024). Enhancing
predictions of acetate and ethanol production from microbial electrosynthesis
using optimized machine learning models. ACS
Sustainable Chem. Eng..

[20] Gadkari S., Fontmorin J. M., Yu E., Sadhukhan J. (2020). Influence
of temperature and other system parameters on microbial fuel cell
performance: Numerical and experimental investigation. Chem. Eng. J..

[21] Perona-Vico E., Blasco-Gómez R., Colprim J., Puig S., Bañeras L. (2019). [NiFe]-hydrogenases
are constitutively expressed in an enriched Methanobacterium sp. population
during electromethanogenesis. PloS one.

[22] Géron, A. Hands-On Machine Learning with Scikit-Learn, Keras, and TensorFlow, 3rd ed.; O’Reilly Media: Sebastopol, CA, 2023.

[23] Chollet, F. , Keras: Deep Learning Library 2015 https://keras.io/

[24] Abadi, M. ; Agarwal, A. ; Barham, P. ; Brevdo, E. ; Chen, Z. ; Citro, C. , TensorFlow: Large scale machine learning on heterogeneous systems 2015 https://www.tensorflow.org/

[25] Pedregosa F., Varoquaux G., Gramfort A., Michel V., Thirion B., Grisel O. (2011). Scikit-learn: Machine learning in Python. J.
Mach. Learn. Res..

[26] Raschka S. (2018). Mlxtend: Providing
machine learning and data science utilities and extensions to Python’s
scientific computing stack. J. Open Source Software.

[27] Gadkari S., Gu S., Sadhukhan J. (2018). Towards automated
design of bioelectrochemical systems:
A comprehensive review of mathematical models. Chem. Eng. J..

[28] Baseer M. A., Singh H., Kumar P., Sperandio Nascimento E. G. (2025). AI-driven
surrogate modelling for simulating hydrogen production via proton
exchange membrane water electrolysers. Int.
J. Hydrogen Energy.

[29] Xiao J., Liu C., Ju B., Xu H., Sun D., Dang Y. (2021). Estimation
of in-situ biogas upgrading in microbial electrolysis cells via direct
electron transfer: Two-stage machine learning modeling based on a
NARX-BP hybrid neural network. Bioresource technology.

[30] Mahapatra D. M., Mishra P., Thakur S., Singh L. (2022). Leveraging artificial
intelligence in bioelectrochemical systems. Trends Biotechnol..

[31] Bolognesi S., Lopez L. R., Perona-Vico E., Bañeras L., Balaguer M. D., Puig S. (2025). Breathe inside the
box: optimizing
conditions for bioelectrochemical methane production to indoor carbon
dioxide valorization and enhance air quality. Chemical Engineering Journal.

[32] Horváth-Gönczi N. N., Bagi Z., Szuhaj M., Rákhely G., Kovács K. L. (2023). Bioelectrochemical systems (BES)
for biomethane production. Fermentation.

[33] Bian Y., Leininger A., May H. D., Ren Z. J. (2024). H2 mediated mixed
culture microbial electrosynthesis for high titer acetate production
from CO2. Environmental Science and Ecotechnology.

[34] Yang H. Y., Bao B. L., Liu J., Qin Y., Wang Y. R., Su K. Z., Han J. C., Mu Y. (2018). Temperature
dependence
of bioelectrochemical CO_2_ conversion and methane production
with a mixed-culture biocathode. Bioelectrochemistry.

[35] Blasco-Gómez R., Batlle-Vilanova P., Villano M., Balaguer M. D., Colprim J., Puig S. (2017). On the edge
of research and technological application: a critical
review of electromethanogenesis. International
journal of molecular sciences.

[36] Sun M., Liu B., Yanagawa K., Ha N. T., Goel R., Terashima M., Yasui H. (2020). Effects of low pH conditions on decay of methanogenic biomass. Water research.

[37] Qiu S., Zhang X., Xia W., Li Z., Wang L., Chen Z., Ge S. (2023). Effect of extreme pH conditions on
methanogenesis: Methanogen metabolism and community structure. Science of The Total Environment.

