# Supplementary material for: Benchmarking Machine Learning Algorithms for Microbial Electromethanogenesis: A Comprehensive Assessment with SHapley Additive exPlanation-Based Insights
Source: ACS Sustain Chem Eng. 2025 Dec 16;14(1):363–75. doi: 10.1021/acssuschemeng.5c09770 (PMC12801386; doi:10.1021/acssuschemeng.5c09770)
Supplement: Supplementary file 1 [file sc5c09770_si_001.pdf]

Supporting Information

Benchmarking Machine Learning Algorithms for Microbial Electro-methanogenesis: A Comprehensive Assessment with SHAP-based Insights

Siddharth Gadkari[a,b,*], Raphael Oliveira[c], Silvia Bolognesi[d], Sebastià Puig[d,*] and Erick Giovani Sperandio Nascimento[b,c,e,f,*]

[a] *School of Chemistry and Chemical Engineering, University of Surrey, Guildford GU2 7XH, United Kingdom*

[b] *Institute for Sustainability, University of Surrey, Guildford GU2 7XH, United Kingdom*

[c] *Stricto Sensu Department, SENAI CIMATEC University, Salvador, Bahia, 41650-010, Brazil*

[d] *LEQUiA. Institute of the Environment, University of Girona, Campus Montilivi. C/Maria Aurelià Capmany, 69, E-17003, Girona, Spain*

[e] *Surrey Institute for People-Centred Artificial Intelligence, Faculty of Engineering and Physical Sciences, University of Surrey, Guildford, GU2 7XH, United Kingdom*

[f] *Global Centre for Clean Research (GCARE), School of Sustainability, Civil and Environmental Engineering and Physical Sciences, University of Surrey, Guildford, GU2 7XH, United Kingdom*

*Corresponding authors

Siddharth Gadkari: s.gadkari@surrey.ac.uk

Sebastià Puig: sebastia.puig@udg.edu

Erick Giovani Sperandio Nascimento: erick.sperandio@surrey.ac.uk

Number of pages: 4

Number of tables: 3

Number of figures: 0

Table S1. Description of the dataset generated for the experimentation.

Dataset	Reactor used	CO_2 feeding strategy	Batch length [d]	Batch characteristics	Current applied [A m^{-2}]
1	EM-MFC	Sampling-feeding	133	Full current range	5-50
2	EM-MFC	Intermittent	42	Intermittent feeding at high current	45
3	EM-EMPC	Sampling-feeding (up to day 82), Intermittent	146	Scaling-up	3-10
4	EM-MFC	Intermittent	136	Powercuts	10-30
5	EM-MFC	Intermittent	136	Powercuts + increased salinity	10-30
6	EM-MFC	Sampling-feeding	41	Control	10-30
7	EM-MFC	Sampling-feeding	41	Batch – NR enhanced	10-30
8	EM-MFC	Sampling-feeding	33	Batch - NR enhanced	10-30
9	EM-MFC	Sampling-feeding	33	Batch - NR enhanced	10-30
10	EM-MFC	Sampling-feeding	18	Batch - NR enhanced	10-30
11	EM-MFC	Sampling-feeding	25	Batch - NR enhanced	10-30

Table S2. Hyperparameters range used in KerasTunner to fine tune the MLP and CNN models.
Legend: Conv1D layer: one-dimensional convolutional layer; Dense layer: One perceptron layer; MAE: Mean Absolute Error ; MSE: Mean Square Error.

Algorithm	Hyperparameters
CNN	Activation function: relu, tanh, leaky relu or gelu
	1 Conv1D layer: filter(min_value=32, max_value=512, step=32)
	1 to 3 blocks:<br>1 Conv1D layer: filter(min_value=32, max_value=512, step=32)<br>1 Conv1D layer: filter(min_value=32, max_value=512, step=32)<br>1 Pooling: max(size=1), average(size=1) or not included<br>1 Dropout layer: range of number [0.0, 0.3], step=0.1
	flatten layer
	1 to 3 blocks:<br>1 Dense layer: range of number of neurons [32, 512], step=32<br>1 Dense layer: range of number of neurons [32, 512], step=32<br>1 Dropout layer: range of number [0.0, 0.3], step=0.1
	1 Dense output layer
	Optimizer: RMSprop or Adam
	Learning rate: 1e-2, 1e-3 or 1e-4
	Metrics: MSE and MAE
	Objective: minimize valuation MSE
MLP	Activation function: relu, tanh, leaky relu or gelu
	1 Dense layer: range of number of neurons [32, 512], step=32
	1 to 4 blocks:<br>1 Dense layer: range of number of neurons [32, 512], step=32<br>1 Dense layer: range of number of neurons [32, 512], step=32

	1 Dropout layer: range of number [0.0, 0.3], step=0.1
	1 Dense output layer
	Optimizer: RMSprop or Adam
	Learning rate: 1e-2, 1e-3 or 1e-4
	Metrics: MSE and MAE
	Objective: minimize valuation MSE

Table S3. Hyperparameters configured for each model used.

Algorithm	Hyperparameters
Adaptive Boosting Regressor	Base estimator: DecisionTreeRegressor with friedman MSE criterion<br>Number of estimators: 500<br>Learning rate: 1
CNN	Input Layer: (1, 5)<br>ConvID Layer: 3<br>Dropout: 0.2<br>ConvID Layer: 2<br>AveragePooling1D: pool_size=1<br>Dropout: 0.1<br>ConvID Layer: 3<br>Dropout: 0.3<br>Flatten<br>Dense Layer: 2<br>Dropout: 0.1<br>Dense Layer: 2<br>Dropout: 0.2<br>Output Layer: (, 1)<br><br>Activation: gelu<br>Optimizer: rmsprop<br>Learning rate: 0.001<br>Figure 3
Gradient Boosting Regressor	Number of estimators: 30,000<br>Max features: sqrt<br>Min samples_split: 10,000<br>Maximum depth: 5<br>Learning rate: 0.1
K-Nearest Neighbor	Number of neighbors: 5
MLP	Input Layer: (, 5)<br>Dense Layer: 3<br>Dropout: 0.2<br>Dense Layer: 2<br>Dropout: 0.1<br>Dense Layer: 2<br>Dropout: 0.2<br>Dense Layer: 2<br>Dropout: 0.1<br>Output Layer: (, 1)<br><br>Activation: gelu<br>Optimizer: rmsprop<br>Figure 4

	Learning rate: 0.01
Stacking Regressor	Regressors: Lasso and Ridge<br>Meta regressor: RandomForestRegressor(n_estimators: 40)<br>Cross validation: 10
Stacking Regressor with Gradient Boosting	Hyperparameters of the Stacking Regressor combined with the hyperparameters of the Gradient Boosting Regressor
